# A Retrospective Evaluation of Perinatal Outcomes in Pregnant Women With Isolated Gestational Proteinuria

**DOI:** 10.7759/cureus.111981

**Published:** 2026-07-03

**Authors:** Berrin Mutlu, İbrahim Kale, Ayse Keleş

**Affiliations:** 1 Obstetrics and Gynecology, Umraniye Training and Research Hospital, Istanbul, TUR; 2 Obstetrics and Gynecology, Maternal Fetal Unit, Umraniye Training and Research Hospital, Istanbul, TUR

**Keywords:** fetal growth restriction, isolated gestational proteinuria, placental abruption, preeclampsia, pregnancy

## Abstract

Objective

This study aimed to examine the role of demographic characteristics and laboratory findings in predicting adverse perinatal outcomes among pregnant women with isolated gestational proteinuria.

Methods

This single-center retrospective study reviewed records of pregnant women diagnosed with gestational proteinuria at the Umraniye Training and Research Hospital, Istanbul, between January 2010 and December 2023. Women with isolated gestational proteinuria were grouped based on subsequent adverse perinatal outcomes. Demographic characteristics and laboratory parameters at the gestational week of initial proteinuria detection were analyzed.

Results

A total of 125 women met the inclusion criteria. Among them, 57 (45.6%) experienced no adverse outcomes, whereas 68 (54.4%) developed at least one complication. Outcomes included preeclampsia (n = 20), isolated fetal growth restriction (FGR) (n = 29), small for gestational age (SGA) (n = 3), isolated preterm birth without concomitant pregnancy-related complications (n = 10), and placental abruption (n = 8), with two cases involving both preeclampsia and subsequent placental abruption. Blood urea nitrogen (BUN) level was significantly higher in the preeclampsia group than in the group without adverse perinatal outcomes (p = 0.025). Proteinuria was detected earlier in gestation in the placental abruption group than in the group without adverse perinatal outcomes (p = 0.012). BUN and serum creatinine levels were significantly higher in the placental abruption group than in the group without adverse perinatal outcomes (p = 0.019 and p = 0.009, respectively).

Conclusions

In women with isolated gestational proteinuria, elevated BUN levels may serve as an early predictor of preeclampsia. Additionally, elevations in BUN and serum creatinine levels may each serve as independent early predictors of placental abruption. Earlier detection of proteinuria appears to be more strongly associated with subsequent placental abruption.

## Introduction

Pregnancy is a unique physiological period for women, marked by significant changes throughout the body. As in other organs, important physiological changes occur in the urinary system during pregnancy. A 75% increase in renal blood flow and a 50% increase in glomerular filtration rate (GFR) in early pregnancy ensure that the GFR remains 50% above the pre-pregnancy level throughout pregnancy [1]. It is considered a normal physiological state for healthy, non-pregnant women to excrete less than 150 mg of protein in urine each day [2]. In healthy pregnancies, the amount of protein excreted in urine can be up to twice that of non-pregnant women. Although increased protein excretion during pregnancy is thought to be due to increased GFR, changes in tubular reabsorption capacity may contribute to increased protein excretion during pregnancy [1].

The amount of protein measured in a 24-hour urine sample is the gold standard for diagnosing proteinuria during pregnancy. However, when an urgent diagnosis is required or when laboratory facilities are insufficient, proteinuria is assessed using a spot urine sample. Accordingly, excess protein of 300 mg or more in a 24-hour urine sample, a urine dipstick result of +1, or a protein/creatinine ratio of 0.3 or more in a spot urine sample is considered diagnostic of proteinuria [1].

The presence of proteinuria together with hypertension after the 20th week of pregnancy is defined as preeclampsia [3]. Preeclampsia complicates 5-10% of pregnancies worldwide. It remains a major cause of maternal and perinatal morbidity and mortality owing to its association with severe complications such as eclampsia, HELLP syndrome, posterior reversible encephalopathy syndrome (PRES), acute kidney injury, placental abruption, fetal growth restriction (FGR), and preterm birth [4,5]. New-onset proteinuria, where blood pressure is normal, and there are no other signs of preeclampsia after the 20th week of pregnancy, is defined as isolated pregnancy proteinuria [1]. The exact incidence of isolated gestational proteinuria is unknown, but it was reported to be 7.7% in a study evaluating 11,651 low-risk pregnant women [6,7]. Although perinatal outcomes of pregnant women with isolated gestational proteinuria seem mostly favorable, it has been reported that up to 30% of women with isolated gestational proteinuria develop preeclampsia in the later weeks of pregnancy [1].

The primary clinical challenge for obstetricians is that it remains unclear which pregnant women with isolated gestational proteinuria will experience an uncomplicated course and which will develop adverse perinatal outcomes. Accordingly, we compared groups of women with isolated gestational proteinuria who developed adverse perinatal outcomes in the later weeks of pregnancy with those who did not, focusing on demographic characteristics and laboratory findings at the time proteinuria was first detected. We aimed to investigate which demographic data or laboratory test results at the gestational week when isolated gestational proteinuria was detected could predict the subsequent development of adverse perinatal outcomes.

## Materials and methods

**Figure 1:**
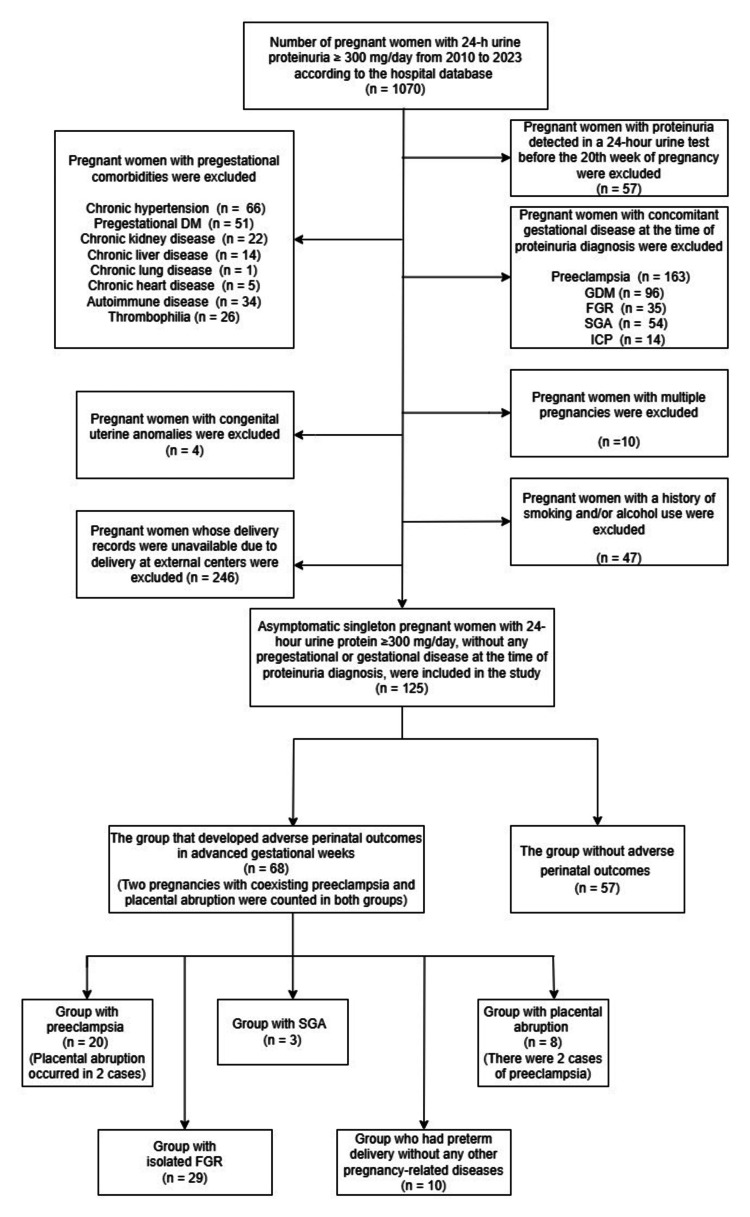
Study flowchart Two pregnancies with placental abruption also developed preeclampsia and were counted in both subgroups. Therefore, the sum of subgroup frequencies exceeds the total number of pregnancies with adverse perinatal outcomes by two cases GDM: gestational diabetes mellitus; FGR: fetal growth restriction; SGA: small for gestational age; ICP: intrahepatic cholestasis of pregnancy

In this single-center retrospective study, data from pregnant women diagnosed with gestational proteinuria between January 2010 and December 2023 at the Umraniye Training and Research Hospital, Department of Obstetrics and Gynecology, Istanbul, Turkey, were collected. In normotensive pregnant women without any underlying disease, the presence of ≥300 mg of protein in a 24-hour urine sample after the 20th week of gestation was defined as isolated gestational proteinuria. Accordingly, women with proteinuria detected before the 20th week of pregnancy were excluded. In addition, pregnant women with any pregestational or gestational comorbidities at the time of proteinuria detection were excluded from the study. Furthermore, smokers, alcohol users, women with congenital uterine anomalies, those with multiple pregnancies, and those who delivered in other hospitals were also excluded. The study flowchart is presented in Figure 1.

Participants were divided into groups based on perinatal outcomes: no adverse perinatal outcomes, preeclampsia, isolated FGR, and placental abruption. The groups were compared with respect to demographic characteristics and laboratory results when isolated gestational proteinuria was detected.

In a normotensive pregnant woman without any pregestational or gestational disease, an excess of 300 mg or more of protein in a 24-hour urine sample after the 20th week of pregnancy was considered isolated gestational proteinuria [1]. The diagnosis of preeclampsia was made according to the American College of Obstetricians and Gynecologists' criteria [3]. Isolated FGR and small for gestational age (SGA) were diagnosed using the criteria reported in the International Society of Ultrasound in Obstetrics and Gynecology (ISUOG) Practice Guidelines [8]. Diagnostic criteria for placental abruption included vaginal bleeding in the second or third trimester, clinical signs of uterine irritability, hypovolemic shock, coagulopathy, or placental abnormalities on sonographic imaging and histologic evidence of abruption [9]. The presence of a retroplacental hematoma confirmed placental abruption in all cases after delivery. Istanbul Umraniye Training and Research Hospital Local Ethics Committee approved this study (approval number: B.10.1.TKH.4.34.H.GP.0.01/34).

Statistical analysis

SPSS Statistics version 27 (IBM Corp., Armonk, NY) was used for statistical analysis. Descriptive statistical methods were used when evaluating the study data. The suitability of quantitative data for normality was assessed using the Shapiro-Wilk test and graphical analysis. The independent t-test, Mann-Whitney U test, and Chi-square test were used to analyze the study data. A receiver operating characteristic (ROC) analysis was used to determine cut-off values for blood urea nitrogen (BUN) and creatinine at the time of isolated gestational proteinuria to predict preeclampsia or placental abruption in advanced weeks of pregnancy. Statistical significance was set at p < 0.05 for all values.

## Results

A total of 1,070 pregnant women underwent 24-hour urine protein testing for various indications and were diagnosed with gestational proteinuria between January 2010 and December 2023. The study included 125 pregnant women who met the eligibility criteria among 1,070 diagnosed with isolated gestational proteinuria. The demographic and clinical characteristics of the 125 included patients are summarized in Table 1.

**Table 1:** Baseline demographic and clinical characteristics of the study population (n = 125) SD: standard deviation; FGR: fetal growth restriction; NICU: neonatal intensive care unit

Variables		Values
Age, years, median (Min–Max)		28 (18–39)
Gravida, median (Min–Max)		2 (1–11)
Parity, median (Min–Max)		1 (0–6)
The gestational week when proteinuria was detected, median (Min–Max)		34.3 (20.0–40.6)
Protein level in the 24-hour urine sample, mg/day, median (Min–Max)		477 (300–8760)
Hemoglobin, g/dL, median (Min–Max)		11.2 (6.3–13.8)
Hematocrit, %, median (Min–Max)		34.1 (21.5–42.4)
Platelet count, 10³/uL, median (Min–Max)		235 (118–515)
Thrombocytopenia, n (%)		7 (5.6%)
Aspartate aminotransferase, u/L, median (Min–Max)		17 (9–95)
Alanine aminotransferase, u/L, median (Min–Max)		11 (4–124)
Lactate dehydrogenase, u/L, median (Min–Max)		226 (129–750)
Blood urea nitrogen, mg/dL, median (Min–Max)		15 (7–34)
Serum creatinine, mg/dL, median (Min–Max)		0.5 (0.3–1.0)
Glomerular filtration rate, mL/min/1.73 m², mean ± SD		130.0 ± 11.5
History of gestational hypertension or preeclampsia, n (%)		13 (10.4%)
History of FGR, n (%)		7 (5.6%)
History of stillbirth, n (%)		6 (4.8%)
Total FGR in the current pregnancy, n (%)		39 (31.2%)
Placental abruption in the current pregnancy, n (%)		8 (6.4%)
Preterm birth in the current pregnancy, n (%)		36 (28.8%)
Emergency cesarean section, n (%)		29 (23.2%)
Gestational week at birth, median (Min–Max)		37.7 (24.3–42.1)
Birth weight, g, median (Min–Max)		2780 (560–4345)
Gender of the newborn, n (%)	Female	66 (52.8%)
	Male	59 (47.2%)
1st minute Apgar score, median (Min–Max)		8 (3–9)
5th minute Apgar score, median (Min–Max)		10 (5–10)
Intrauterine fetal death, n (%)		4 (3.2%)
NICU admission, n (%)		28 (22.4%)

While 57 (45.6%) of the 125 pregnant women with isolated gestational proteinuria did not develop any adverse perinatal outcomes, 68 (54.4%) of them had adverse perinatal outcomes. Preeclampsia occurred in 20, isolated FGR in 29, SGA in three, isolated preterm birth without concomitant pregnancy-related complications in 10, and placenta abruption in eight (preeclampsia and subsequent placental abruption developed in two pregnant women).

The preeclampsia group and the group without adverse perinatal outcomes were compared for demographic characteristics and laboratory results. Age, gravida, parity, gestational week at which isolated proteinuria was detected, and 24-hour urine protein level were similar in both groups (p > 0.05 for all comparisons). Both groups were similar in hemoglobin, hematocrit, platelet count, number of pregnant women with thrombocytopenia, aspartate aminotransferase (AST), alanine aminotransferase (ALT), lactate dehydrogenase (LDH), serum creatinine, and GFR (p > 0.05 for all comparisons). BUN level was significantly higher in the preeclampsia group than in the group without adverse perinatal outcomes (p = 0.025). Both groups were similar in terms of history of gestational hypertension or preeclampsia in previous pregnancies, history of FGR, and history of stillbirth (p > 0.05 for all) (Table 2).

**Table 2:** Comparison of demographic characteristics, laboratory findings, and perinatal outcomes between the preeclampsia group and the group without adverse perinatal outcomes ^a^Mann-Whitney U test. ^B^Independent t-Test. ^c^Chi-square test SD: standard deviation; FGR: fetal growth restriction; NICU: neonatal intensive care unit

Variables		Preeclampsia group (n = 20)	Group without adverse perinatal outcomes (n = 57)	Test statistic	P-value
Age, years, median (Q1-Q3)		28.5 (25.0–32.0)	27.0 (25.0–33.0)	U = 592	0.798^a^
Gravida, median (Q1-Q3)		3.0 (2.0–3.2)	2.0 (1.0–3.0)	U = 697	0.129^a^
Parity, median (Q1-Q3)		1.0 (0.8–2.0)	1.0 (0.0–2.0)	U = 640	0.393^a^
The gestational week when proteinuria was detected, median (Q1–Q3)		34.4 (31.9–35.2)	34.9 (32.0–37.3)	U = 451.5	0.169^a^
Protein level in the 24-hour urine sample, mg/day, median (Q1–Q3)		549.0 (396.8-870.8)	460.0 (354.0–653.0)	U = 666.5	0.262^a^
Hemoglobin, g/dL, median (Q1–Q3)		11.1 (10.4–11.7)	11.2 (10.1–12.4)	U = 546.5	0.785^a^
Hematocrit, %, median (Q1–Q3)		34.7 (31.7–36.3)	33.8 (31.5–36.8)	U = 566.5	0.968^a^
Platelet count, 10³/uL, median (Q1–Q3)		223.5 (192.8–269.5)	221.0 (191.0–289.0)	U = 557	0.880^a^
Thrombocytopenia, n (%)	Yes	1 (5)	3 (5.3)	X^2^ = 0.000	0.724^c^
	No	19 (95)	54 (94.7)		
Aspartate aminotransferase, u/L, median (Q1–Q3)		16.0 (13.5–25.0)	17.0 (13.0–23.0)	U = 568	0.981^a^
Alanine aminotransferase, u/L, median (Q1–Q3)		10.5 (8.8–15.2)	12.0 (9.0–14.0)	U = 530.5	0.645^a^
Lactate dehydrogenase, u/L, median (Q1–Q3)		224.0 (199.0–306.0)	209.0 (180.0–304.0)	U = 646	0.377^a^
Blood urea nitrogen, mg/dL, median (Q1–Q3)		17.0 (12.0–23.0)	14.0 (11.0–19.0)	U = 762.5	0.025^a^
Serum creatinine, mg/dL, median (Q1–Q3)		0.6 (0.5–0.7)	0.5 (0.4–0.6)	U = 680.5	0.199^a^
Glomerular filtration rate, mL/min/1.73 m², mean ± SD		126.7 ± 12.1	131 ± 12.8	t = -1.320	0.191^b^
History of gestational hypertension or preeclampsia, n (%)	Yes	5 (25)	5 (8.8)	X^2^ = 2.164	0.075^c^
	No	15 (75)	52 (91.2)		
History of FGR, n (%)	Yes	3 (15)	1 (1.8)	X^2^ = 2.928	0.052^c^
	No	17 (85)	56 (98.2)		
History of stillbirth, n (%)	Yes	1 (5)	1 (1.8)	X^2^ = 0.000	0.455^c^
	No	19 (95)	56 (98.2)		
FGR in the current pregnancy, n (%)	Yes	10 (50)	0 (0)	X^2^ = 28.479	< 0.001^c^
	No	10 (50)	57 (100)		
Placental abruption in the current pregnancy, n (%)	Yes	2 (10)	0 (0)	X^2^ = 2.567	0.065^c^
	No	18 (90)	57 (100)		
Preterm birth in the current pregnancy, n (%)	Yes	12 (60)	0 (0)	X^2^ = 36.081	< 0.001^c^
	No	8 (40)	57 (100)		
Emergency cesarean section, n (%)	Yes	9 (45)	6 (10.5)	X^2^ = 9.127	0.002^c^
	No	11 (55)	51 (89.5)		
Gestational week at birth, median (Q1–Q3)		36.6 (34.9–37.5)	38.6 (38.0–39.4)	U = 153	< 0.001^a^
Birth weight, g, median (Q1–Q3)		2365.0 (2047.5–2757.5)	3240.0 (2890.0–3560.0)	U = 144	< 0.001^a^
Gender of the newborn, n (%)	Female	9 (45)	31 (54.4)	X^2^ = 0.214	0.322^c^
	Male	11 (55)	26 (45.6)		
1st minute Apgar score, median (Q1–Q3)		8.0 (8.0–9.0)	9.0 (8.0–9.0)	U = 450	0.110^a^
5th minute Apgar score, median (Q1–Q3)		9.5 (9.0–10.0)	10.0 (9.0–10.0)	U = 464	0.148^a^
Intrauterine fetal death, n (%)	Yes	0 (0)	0 (0)	-	-
	No	20 (100)	57 (100)		
NICU admission, n (%)	Yes	7 (35)	3 (5.3)	X^2^ = 9.103	0.002^c^
	No	13 (65)	54 (94.7)		

**Figure 2:**
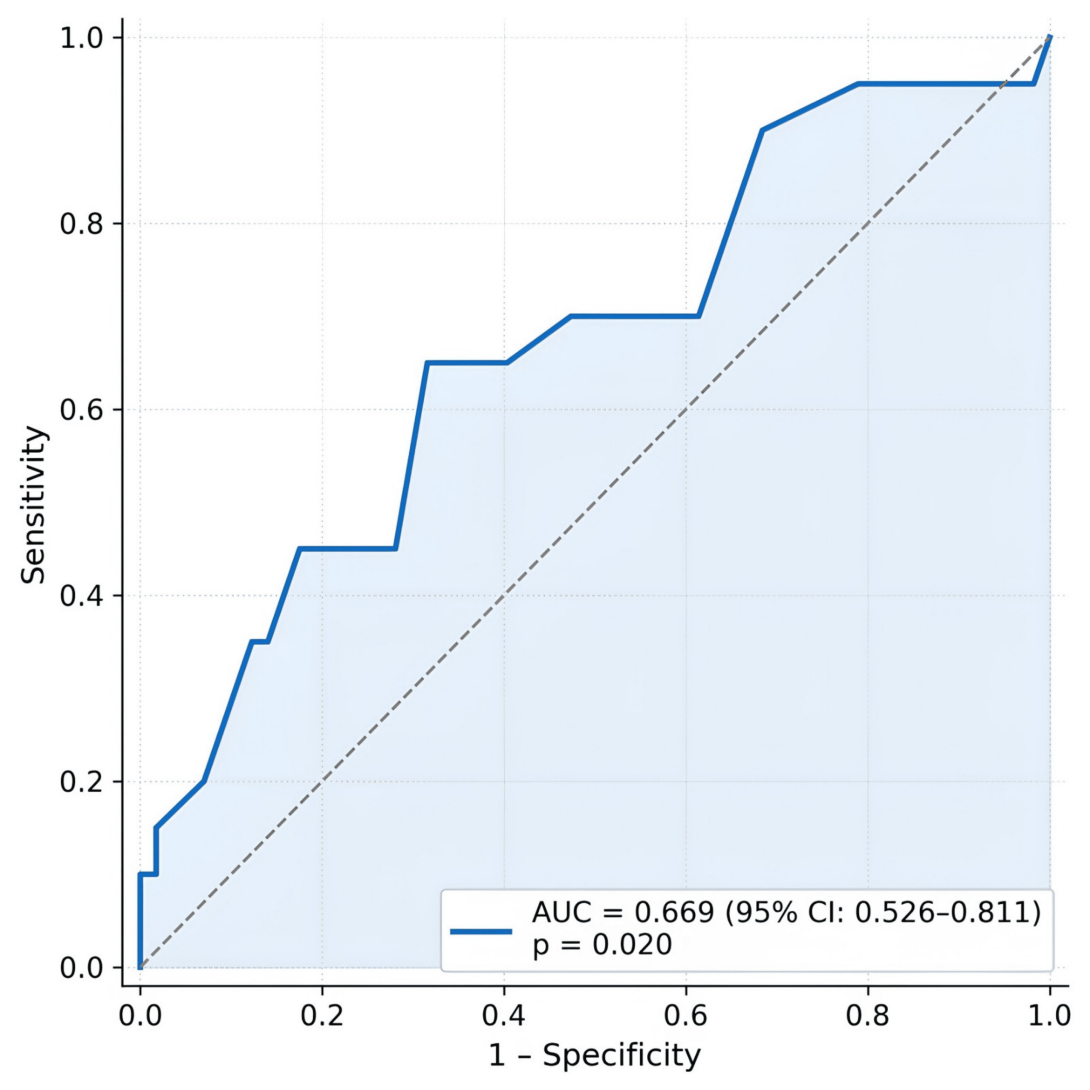
ROC analysis of BUN measured at the time of isolated gestational proteinuria for predicting preeclampsia in later weeks of gestation ROC: receiver operating characteristic; BUN: blood urea nitrogen; AUC: area under the curve; CI: confidence interval

A ROC analysis was conducted to determine the BUN cutoff value for predicting preeclampsia in the later weeks of pregnancy. Accordingly, the level of 16.5 mg/dL of BUN at the time of isolated proteinuria detection had a sensitivity of 65% and a specificity of 68.4% (area under the curve (AUC): 0.669, 95% confidence interval (CI): 0.52 - 0.81), indicating the risk of preeclampsia in the later weeks of pregnancy (Figure 2).

The isolated FGR group and the group without adverse perinatal outcomes were compared with respect to demographic characteristics and laboratory results. Age, gravida, parity, gestational age at which isolated proteinuria was detected, and 24-hour urine protein level were similar between the groups (p > 0.05 for all comparisons). Both groups were similar in hemoglobin, hematocrit, platelet count, number of pregnant women with thrombocytopenia, AST, ALT, LDH, BUN, serum creatinine, and GFR (p > 0.05 for all comparisons). Both groups were similar in terms of history of gestational hypertension or preeclampsia in previous pregnancies, history of FGR, and history of stillbirth (p > 0.05 for all) (Table 3).

**Table 3:** Comparison of demographic characteristics, laboratory findings, and perinatal outcomes between the isolated FGR group and the group without adverse perinatal outcomes ^a^Mann-Whitney U test. ^B^Independent t-Test. ^c^Chi-square test SD: standard deviation; FGR: fetal growth restriction; NICU: neonatal intensive care unit

Variables		Isolated FGR group (n = 29)	Group without adverse perinatal outcomes (n = 57)	Test statistic	P-value
Age, years, median (Q1-Q3)		28 (24–32)	27 (25–33)	U = 783.5	0.694^a^
Gravida, median (Q1-Q3)		2 (1–3)	2 (1–3)	U = 726	0.341^a^
Parity, median (Q1-Q3)		1 (0–1)	1 (0–2)	U = 782.5	0.671^a^
The gestational week when proteinuria was detected, median (Q1-Q3)		34 (30.7–36.3)	34.9 (32–37.3)	U = 748.5	0.476^a^
Protein level in the 24-hour urine sample, mg/day, median (Q1-Q3)		477 (414–958)	460 (354–653)	U = 941.5	0.293^a^
Hemoglobin, g/dL, median (Q1-Q3)		11.4 (11–12.5)	11.2 (10.1–12.4)	U = 1013	0.088^a^
Hematocrit, %, median (Q1-Q3)		35.5 (33.3–36.7)	33.8 (31.5–36.8)	U = 947.5	0.269^a^
Platelet count, 10³/uL, median (Q1-Q3)		236 (216–258)	221 (191–289)	U = 873	0.671^a^
Thrombocytopenia, n (%)	Yes	1 (3.4)	3 (5.3)	X^2^ = 0.000	0.586^c^
	No	28 (96.6)	54 (94.7)		
Aspartate aminotransferase, u/L, median (Q1-Q3)		16 (13–18)	17 (13–23)	U = 775.5	0.641^a^
Alanine aminotransferase, u/L, median (Q1-Q3)		11 (9–13)	12 (9–14)	U = 787.5	0.721^a^
Lactate dehydrogenase, u/L, median (Q1-Q3)		267 (198–401)	209 (180–304)	U = 1017.5	0.081^a^
Blood urea nitrogen, mg/dL, median (Q1-Q3)		17 (13–20)	14 (11–19)	U = 1007.5	0.097^a^
Serum creatinine, mg/dL, median (Q1-Q3)		0.6 (0.4–0.6)	0.5 (0.4–0.6)	U = 888	0.574^a^
Glomerular filtration rate, mL/min/1.73 m², mean ± SD		129.6 ± 10.8	131 ± 12.8	t = -0.490	0.625^b^
History of gestational hypertension or preeclampsia, n (%)	Yes	0 (0)	5 (8.8)	X^2^ = 1.337	0.120^c^
	No	29 (100)	52 (91.2)		
History of FGR, n (%)	Yes	2 (6.9)	1 (1.8)	X^2^ = 0.369	0.262^c^
	No	27 (93.1)	56 (98.2)		
History of stillbirth, n (%)	Yes	3 (10.3)	1 (1.8)	X^2^ = 1.555	0.109^c^
	No	26 (89.7)	56 (98.2)		
Preterm birth in the current pregnancy, n (%)	Yes	9 (31)	0 (0)	X^2^ = 16.584	< 0.001^c^
	No	20 (69)	57 (100)		
Emergency cesarean section, n (%)	Yes	10 (34.5)	6 (10.5)	X^2^ = 5.788	0.007^c^
	No	19 (65.5)	51 (89.5)		
Gestational week at birth, median (Q1-Q3)		37.4 (35.4–38.6)	38.6 (38–39.4)	U = 401	<0.001^a^
Birth weight, g, median (Q1-Q3)		2180 (1790–2730)	3240 (2890–3560)	U = 176.5	<0.001^a^
Gender of the newborn, n (%)	Female	16 (55.2)	31 (54.4)	X^2^ = 0.000	0.564^c^
	Male	13 (44.8)	26 (45.6)		
1st minute Apgar score, median (Q1-Q3)		8 (8–8)	9 (8–9)	U = 430	0.002^a^
5th minute Apgar score, median (Q1-Q3)		9 (9–10)	10 (9–10)	U = 524	0.028^a^
Intrauterine fetal death, n (%)	Yes	4 (13.8)	0 (0)	X^2^ = 5.429	0.011^c^
	No	25 (86.2)	57 (100)		
NICU admission, n (%)	Yes	11 (44)	3 (5.3)	X^2^ = 15.784	<0.001^c^
	No	14 (56)	54 (94.7)		

The placental abruption group and the group without adverse perinatal outcomes were compared for demographic characteristics and laboratory results. Age, gravida, parity, and 24-hour urine protein level were similar between the two groups (p > 0.05 for all comparisons). The week of proteinuria detection was significantly lower in the placental abruption group than in the group without adverse perinatal outcomes (p = 0.012). Both groups were similar in hemoglobin, hematocrit, platelet count, number of pregnant women with thrombocytopenia, AST, ALT, LDH, and GFR (p > 0.05 for all comparisons). BUN and serum creatinine values ​​were found to be significantly higher in the placental abruption group compared to the group without adverse perinatal outcomes (p = 0.019, p = 0.009, respectively). Both groups were similar in terms of history of gestational hypertension or preeclampsia in previous pregnancies, history of FGR, and history of stillbirth (p > 0.05 for all) (Table 4).

**Table 4:** Comparison of demographic characteristics, laboratory findings, and perinatal outcomes between the placental abruption group and the group without adverse perinatal outcomes ^a^Mann-Whitney U test. ^B^Independent t-test. ^c^Chi-square test SD: standard deviation; FGR: fetal growth restriction; NICU: neonatal intensive care unit

Variables		Placental abruption group (n = 8)	Group without adverse perinatal outcomes (n = 57)	Test statistic	P-value
Age, years, median (Q1-Q3)		29.5 (26.5–32.5)	27 (25–33)	U = 253.5	0.609^a^
Gravida, median (Q1-Q3)		2 (2–3.2)	2 (1–3)	U = 253	0.607^a^
Parity, median (Q1-Q3)		1 (0.8–2.2)	1 (0–2)	U = 271.5	0.362^a^
The gestational week when proteinuria was detected, median (Q1-Q3)		31.1 (29–32.1)	34.9 (32–37.3)	U = 101.5	0.012^a^
Protein level in the 24-hour urine sample, mg/day, median (Q1-Q3)		1375.5 (413–1675)	460 (354–653)	U = 314	0.086^a^
Hemoglobin, g/dL, median (Q1-Q3)		12 (11.0–12.9)	11.2 (10.1–12.4)	U = 281	0.290^a^
Hematocrit, %, median (Q1-Q3)		36 (34.2–37.0)	33.8 (31.5–36.8)	U = 262.5	0.491^a^
Platelet count, 10³/uL, median (Q1-Q3)		227 (200.5–244.5)	221 (191–289)	U = 212.5	0.757^a^
Thrombocytopenia, n (%)	Yes	1 (12.5)	3 (5.3)	X^2^ = 0.000	0.417^c^
	No	7 (87.5)	54 (94.7)		
Aspartate aminotransferase, u/L, median (Q1-Q3)		16.5 (14.8–23.2)	17 (13–23)	U = 235	0.889^a^
Alanine aminotransferase, u/L, median (Q1-Q3)		11 (9.5–21.2)	12 (9–14)	U = 247.5	0.696^a^
Lactate dehydrogenase, u/L, median (Q1-Q3)		277 (236.2–304)	209 (180–304)	U = 264.5	0.466^a^
Blood urea nitrogen, mg/dL, median (Q1-Q3)		22 (16.5–26.2)	14 (11–19)	U = 345	0.019^a^
Serum creatinine, mg/dL, median (Q1-Q3)		0.6 (0.6–0.7)	0.5 (0.4–0.6)	U = 358	0.009^a^
Glomerular filtration rate, mL/min/1.73 m², mean ± SD		124 ± 11.7	131 ± 12.8	t = -1.457	0.150^b^
History of gestational hypertension or preeclampsia, n (%)	Yes	0 (0)	5 (8.8)	X^2^= 0.027	0.507^c^
	No	8 (100)	52 (91.2)		
History of FGR, n (%)	Yes	1 (12.5)	1 (1.8)	X^2^ = 0.308	0.233^c^
	No	7 (87.5)	56 (98.2)		
History of stillbirth, n (%)	Yes	1 (12.5)	1 (1.8)	X^2^ = 0.308	0.233^c^
	No	7 (87.5)	56 (98.2)		
Preterm birth in the current pregnancy, n (%)	Yes	7 (87.5)	0 (0)	X^2^ = 47.160	< 0.001^c^
	No	1 (12.5)	57 (100)		
FGR in the current pregnancy, n (%)	Yes	2 (25)	0 (0)	X^2^ = 7.514	0.013^c^
	No	6 (75)	57 (100)		
Preeclampsia in the current pregnancy, n (%)	Yes	2 (25)	0 (0)	X^2^ = 7.514	0.013^c^
	No	6 (75)	57 (100)		
Emergency cesarean section, n (%)	Yes	8 (100)	6 (10.5)	X^2^ = 28.149	< 0.001^c^
	No	0 (0)	51 (89.5)		
Gestational week at birth, median (Q1-Q3)		31.8 (30.8–34.8)	38.6 (38–39.4)	U = 54.5	0.001^a^
Birth weight, g, median (Q1-Q3)		1445 (1267.5–1950)	3240 (2890–3560)	U = 51.5	< 0.001
Gender of the newborn, n (%)	Female	4 (50)	31 (54.4)	X^2^ = 0.000	0.554^c^
	Male	4 (50)	26 (45.6)		
1st minute Apgar score, median (Q1-Q3)		4 (3.5–7)	9 (8–9)	U = 76.5	0.002^a^
5th minute Apgar score, median (Q1-Q3)		6 (6–8.5)	10 (9–10)	U = 76	0.002^a^
Intrauterine fetal death, n (%)	Yes	1 (12.5)	0 (0)	X^2^ = 1.337	0.123^c^
	No	7 (87.5)	57 (100)		
NICU admission, n (%)	Yes	6 (85.7)	3 (5.3)	X^2^ = 27.064	< 0.001^c^
	No	1 (14.3)	54 (94.7)		

**Figure 3:**
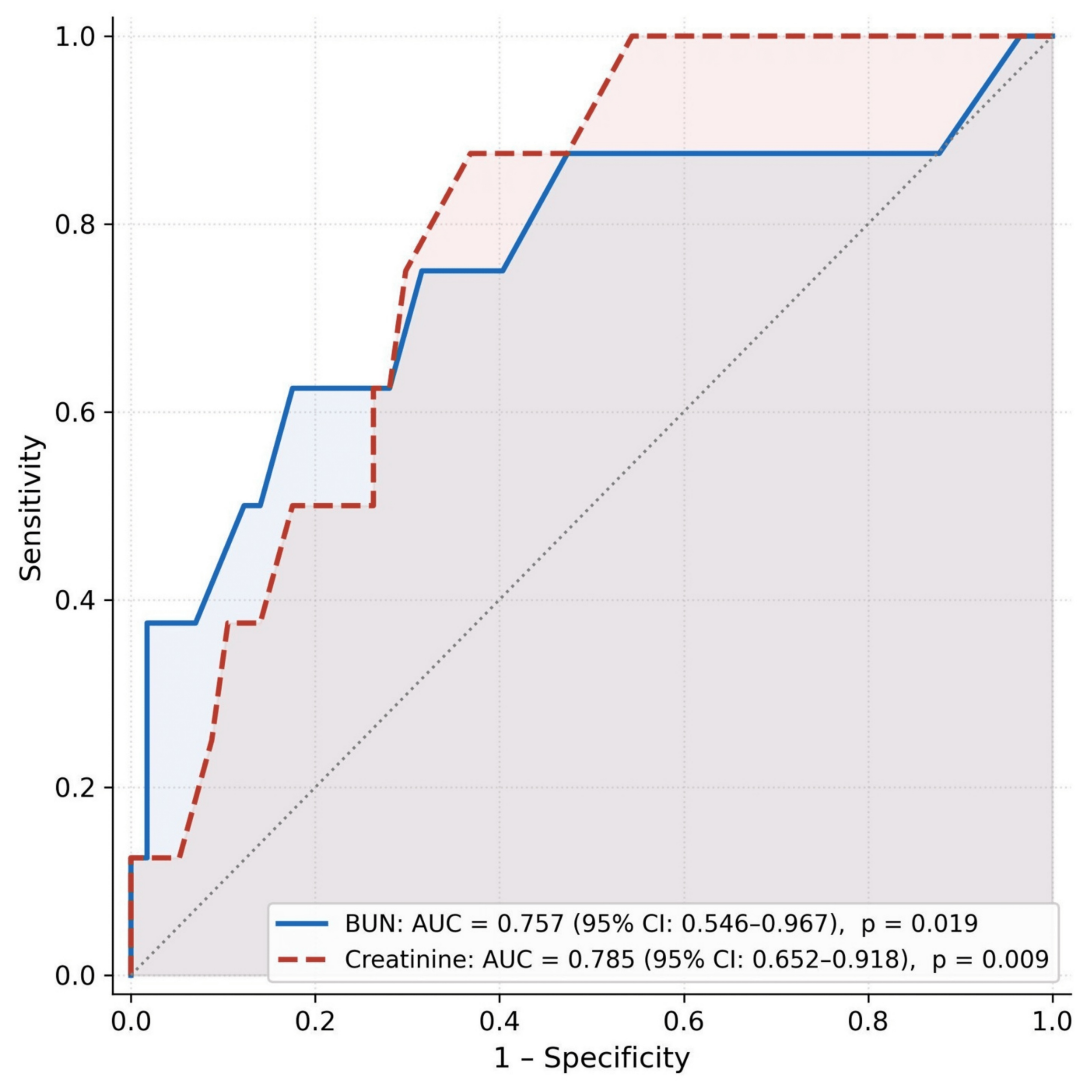
ROC analysis of BUN and serum creatinine measured at the time of isolated gestational proteinuria for predicting placental abruption in later weeks of gestation ROC: receiver operating characteristic; BUN: blood urea nitrogen; AUC: area under the curve; CI: confidence interval

A ROC analysis was conducted to determine the cutoff value of BUN and serum creatinine that can be used to predict the development of placental abruption in the later weeks of pregnancy. Accordingly, BUN at 16.5 mg/dL at the time of detection of isolated proteinuria predicted placental abruption in advanced weeks of gestation with 75% sensitivity and 68.4% specificity (AUC: 0.757, 95% CI: 0.54 - 0.96) and serum creatinine at 0.56 mg/dL predicted placental abruption with 75% sensitivity and 70.2% specificity (AUC: 0.785, 95% CI: 0.65 - 0.91) (Figure 3).

## Discussion

Adverse perinatal outcomes following the diagnosis of isolated gestational proteinuria have been investigated in many different studies. The literature reports varying rates of preeclampsia development in pregnant women with isolated proteinuria in the advanced or subsequent weeks of pregnancy, ranging from 22.1% to 33.7% [7,10,11,14]. In our study, the rate of preeclampsia development in pregnant women with isolated proteinuria was found to be 16%.

In the studies, clinical characteristics and laboratory findings of pregnant women who developed preeclampsia after the diagnosis of isolated proteinuria were compared with those of women who remained normotensive until delivery. Chung et al. reported that age, primiparity, gestational week at which proteinuria was detected, and proteinuria levels were similar between groups with and without preeclampsia after the diagnosis of isolated gestational proteinuria. A history of prior preeclampsia was found to be significantly higher in the group that developed preeclampsia than in the group that did not [11]. In another study, the protein concentration in the 24-hour urine sample was significantly higher in the group that developed preeclampsia than in the group that did not. In contrast, the gestational age at which proteinuria was detected was lower [10].

Shinar et al. compared the group with isolated gestational proteinuria who developed preeclampsia with the group that did not, and both groups were similar in age, primiparity, proteinuria level, and gestational age at which proteinuria was detected [14]. Erkenekli et al. compared groups with isolated gestational proteinuria who developed preeclampsia with those who did not. Both groups were similar in age, parity, gestational week at which proteinuria was detected, proteinuria amount, serum creatinine level, AST level, and platelet count [15]. In this study, we found that age, parity, gestational week at which proteinuria was detected, amount of proteinuria, serum creatinine level, AST level, and platelet count were similar between the groups that developed and did not develop preeclampsia. However, the BUN was significantly higher in the preeclampsia group than in the group without adverse perinatal outcomes.

A 2021 study demonstrated that increasing levels of isolated gestational proteinuria are associated with a higher risk of developing preeclampsia with severe features in pregnant women after 24 weeks of gestation. The study also reported a clear dose-response relationship, with the risk of severe preeclampsia increasing progressively across higher proteinuria levels [12]. Similarly, a recent study showed that among women with new-onset isolated proteinuria after 24 weeks of gestation, marked proteinuria is significantly associated with an increased risk of preeclampsia, particularly with severe features, as well as adverse perinatal outcomes [13].

Although several studies on isolated gestational proteinuria have been published, few have examined its relationship to the development of FGR. In a study by Stettler et al., the rate of FGR in the advanced weeks of pregnancy was 23% among those with gestational proteinuria [16]. In another study published in 2024, 61 pregnant women with isolated gestational proteinuria were evaluated in terms of perinatal outcomes, and 34 (55.7%) of them developed FGR [17]. In our study, the rate of isolated FGR development in pregnant women with isolated proteinuria was 23.2%. Also, when we compared the isolated FGR group with the group without adverse perinatal outcomes with respect to demographic characteristics and laboratory findings at the time of diagnosis of isolated gestational proteinuria, we found no differences between the two groups.

We identified only a single study in the literature examining the association between isolated gestational proteinuria and placental abruption. In this paper, it was reported that the risk of placental abruption increases as the degree of proteinuria increases [12]. In our study, six out of eight cases diagnosed with isolated gestational proteinuria and experiencing placental abruption in the later weeks of pregnancy were normotensive. In the group with placental abruption, proteinuria was detected significantly earlier in pregnancy compared with the group without adverse perinatal outcomes, while the amount of proteinuria was comparable between the two groups. Also, it was observed that BUN and serum creatinine levels were significantly higher in the placental abruption group than in the group without adverse perinatal outcomes.

This study has several important limitations. The relatively small sample size in the study groups and the absence of data for key clinical variables, such as BMI, uric acid levels, and aspirin use, represent notable constraints of this single-center, retrospective design. In addition, because the study was retrospective, biomarkers such as D-dimer and fibrin monomer were not routinely measured. Therefore, they could not be evaluated, potentially limiting the assessment of early coagulation abnormalities.

## Conclusions

In pregnant women diagnosed with isolated gestational proteinuria, high BUN levels may be an early indicator of preeclampsia. Additionally, levels may serve as early indicators of placental abruption. Furthermore, the earlier isolated gestational proteinuria is detected during pregnancy, the more it appears to be associated with placental abruption.
